# Correlation between First and Second Trimester Uterine Artery Doppler Velocimetry and Placental Bed Histopathology

**DOI:** 10.1155/2014/890534

**Published:** 2014-07-13

**Authors:** Murat Akbaş, Cihat Şen, Zerrin Calay

**Affiliations:** ^1^Department of Obstetrics and Gynecology, Okmeydani Research Hospital, 34382 Istanbul, Turkey; ^2^Department of Obstetrics and Gynecology, Cerrahpasa Faculty of Medicine, Istanbul University, 34098 Istanbul, Turkey; ^3^Department of Pathology, Cerrahpasa Faculty of Medicine, Istanbul University, 34098 Istanbul, Turkey

## Abstract

*Aim*. To evaluate the relationship between uterine artery Doppler indices and placental bed histopathology independent of clinical outcome. *Materials and Methods*. Uterine artery measurements were performed to 510 pregnant women who had come for routine antenatal care in 11–14th and 20–24th weeks. Placental bed biopsies from 141 cases were taken during cesarean section. Physiological changes and abnormal placental histology findings were investigated and compared with Doppler findings. *Results*. 116 biopsies were accepted as adequate biopsy and included in the study. Physiological changes were seen in 100 biopsies. Statistically significant higher PI and RI values in second trimester and higher notch rate in both trimesters were detected in the abnormal placental histology group (*P* < 0,001). *Conclusion*. Strong relationship between uterine artery Doppler indices and preeclampsia or intrauterine growth retardation has been shown in previous studies. In our study, we concluded that there is significant relationship between Doppler findings and placental bed histopathology independent of clinical course.

## 1. Introduction

Spiral arteries change into loose vessels for providing increased blood flow to fetus during pregnancy. These changes in uteroplacental vessels were identified by Brosens et al. and named “physiological changes” in 1967 [1]. Spiral artery remodelling begins before cellular interaction with trophoblasts. The first changes in spiral arteries involve endothelial vacuolation, arterial dilation, and smooth muscle disorganization. Similar structural alterations were seen in ectopic pregnancies and in the decidua parietalis of intrauterine pregnancies [2]. Decomposition of arterial smooth muscle and fibrinoid material accumulation are seen when trophoblasts begin to appear near arteries [3]. Trophoblasts settle in this fibrinoid structure and take place of smooth muscle cells [4]. Trophoblasts change their adhesion molecules before invading the spiral arteries [5]. Oxygen tension determines whether cytotrophoblasts proliferate or invade, thereby regulating placental growth and cellular architecture. At early gestation, trophoblasts form plugs in the maternal spiral arteries occluding the flow of maternal blood to the intervillous space [6–8]. Thus, trophoblasts have proliferative characteristics under low oxygen pressure [9].

Trophoblasts replace maternal endothelium as far as the inner third of the myometrium. Placental bed biopsies of pregnancies that had been complicated with preeclampsia or intrauterine growth retardation (IUGR) showed inadequate trophoblastic invasion on myometrial component of spiral arteries [10]. Studies showed that defective invasion is not only seen on myometrial segment, but also decidual [11]. In addition, subintimal thickening, fibrinoid necrosis, acute thrombosis, perivascular nuclear cell infiltration in spiral arteries, and trophoblastic giant cells in stroma are found in preeclampsia and IUGR cases [12, 13]. Defective placentation is an important predisposing factor and endothelial injury is associated with signs of these diseases [14–17].

First and second trimester uterine artery Doppler assessments have high predictive value for clinical outcome (prediction of preeclampsia and IUGR) [18]. Studies have shown that, in pregnancies which are complicated with preeclampsia and/or IUGR, fetal and uterine artery Doppler indices are related to placental bed biopsy histopathology [19, 20]. In these studies, Doppler measurements were performed after preeclampsia and/or IUGR clinic had been emerged and biopsies were taken during cesarean section.

In studies which have correlated Doppler velocimetry and clinical outcomes, wide range of specificity and sensitivity values were found [18]. High resistance findings in Doppler velocimetry are signs of defective placentation, but clinical disease has multifactorial pathophysiology. The aim of the present study is to correlate the histopathology of the placental bed and first and second trimester Doppler velocimetry of uterine arteries.

## 2. Material and Method

### 2.1. Study Design

A prospective study of women who attended for antenatal care at 11–14th and 20–24th weeks of gestation was constructed from a cohort of 510 women with singleton pregnancies who was followed up in Istanbul University Cerrahpasa Medical Faculty Hospital, Perinatology Department, Turkey, between January 2010 and July 2011. 270 cases delivered in our clinic and 141 cases delivered by cesarean section and were included in the study. Cases who were complicated with essential hypertension, diabetes mellitus, and collagen vascular diseases were excluded because these diseases' pathological findings are classified separately. Approval for the study was given by Istanbul Clinical Research Ethical Committee and informed consent was obtained.

### 2.2. Doppler Ultrasonography and Sample Collection

In 11–14th and 20–24th weeks of gestation during routine antenatal screening bilateral uterine artery Doppler measurements were performed. All measurements were performed with the mothers in a semirecumbent position. Colour-flow imaging was used to visualize the ascending branch of the uterine arteries. Pulsed Doppler velocimetry was performed with a sample volume of 5 mm. Two measurements were made from each artery and mean value was calculated.

During cesarean section after removal of placenta and membranes, biopsies were taken from placental bed side by palpation with Kevorkian punch biopsy tool. 3-4 *μ*m paraffin sections were obtained from placental bed biopsy specimens. After staining with hematoxylin-eosin, cytokeratin 7, and periodic acid schiff, specimens were examined under light microscopy. All placental bed biopsies were examined by the same pathologist who was blinded to all clinical data.

Biopsies are accepted as histologically enough if they

1. are placental bed biopsy (contain trophoblast),
2. contain spiral artery,
3. contain myometrial component.

Biopsy specimens were classified as “normal histology” if the histological features of the normal human placental bed (endovascular trophoblast invasion in decidual and myometrial segment, normal spiral artery appearance) were documented or “abnormal placental histology” if there were inadequate response to placentation, acute atherosis, increased extravillous trophoblasts, and thrombosis or luminal obliteration of spiral arteries.

### 2.3. Clinical Definitions

Intrauterine growth restriction was diagnosed when fetal abdominal circumference was more than two standard deviations below the mean for gestational age and also confirmed by the serial assessment of the fetal growth parameters. Preeclampsia was defined as the onset of hypertension (blood pressure of 140/90 mmHg or greater, 6 or more hours apart) and consistent proteinuria (>300 mg/day) during the latter half of the pregnancy and both remitting remotely after delivery. Oligohydramnios was diagnosed when amniotic fluid index was less than 5 cm.

### 2.4. Statistics

Data analysis was made by a personal computer with SPSS 16.0 statistical program. Demographic and clinical characteristics of the study groups were compared using Pearson's chi-square test. Student's *t*-test or Mann-Whitney *U*-test was used for comparison of two groups where appropriate. A *P* value <0.05 was considered significant.

## 3. Results

**Figure 1:**
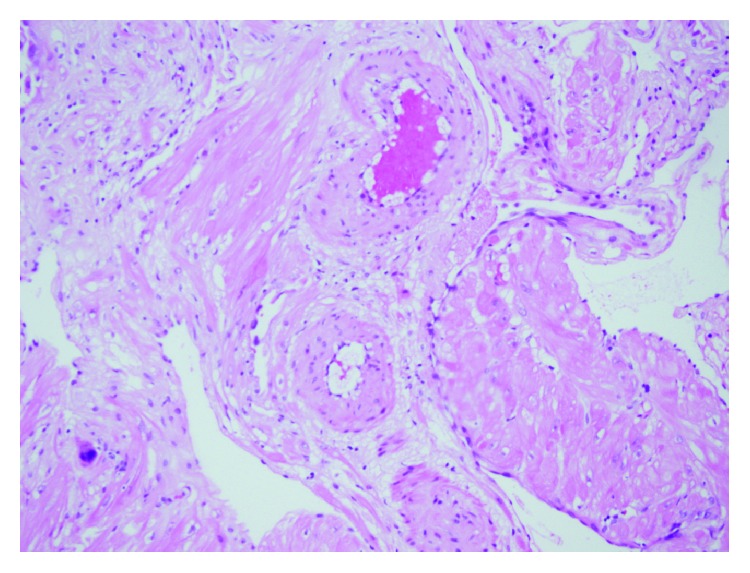
Acute atherosis with lipid laden macrophages in lumens of spiral arteries.

**Figure 2:**
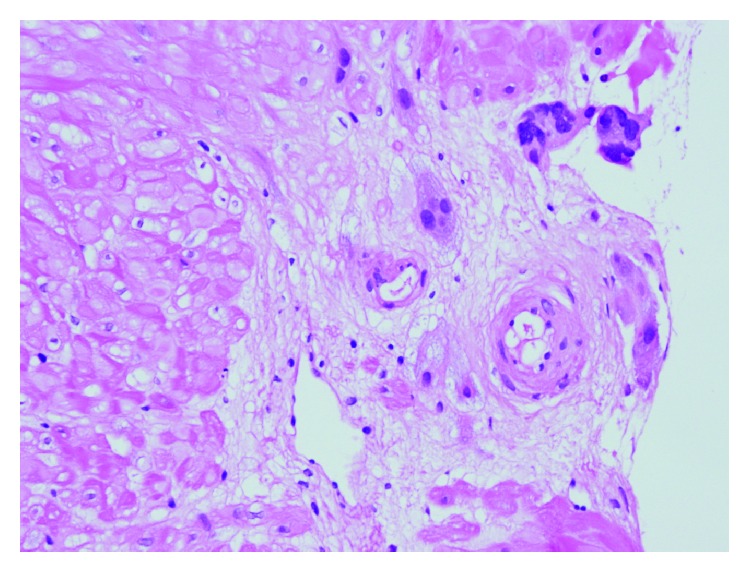
Deficient spiral artery remodelling and increased extravillous trophoblasts around arteries.

**Figure 3:**
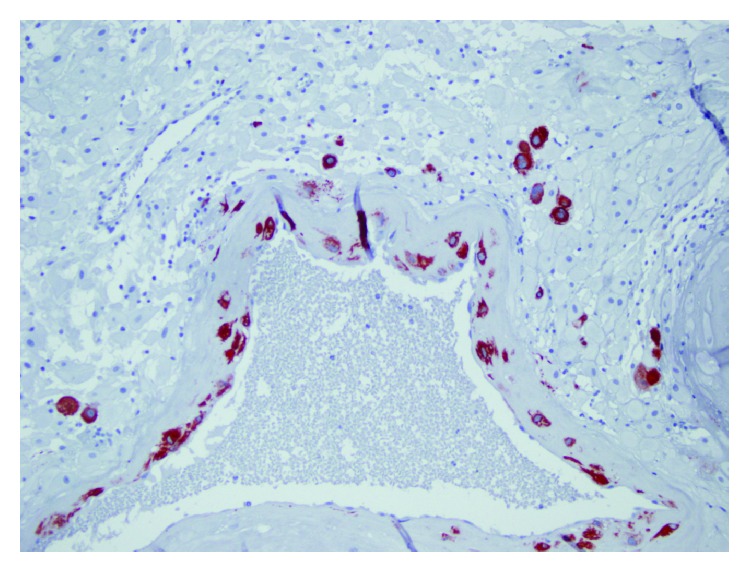
Spiral artery with physiological change and cytokeratin-7-positive trophoblasts in the arterial wall.

Indications for cesarean delivery were prior cesarean section (76/141), cephalopelvic disproportion (36/141), breech presentation (17/141), and fetal distress during labor (12/141). Successful biopsy rate was 82,2% (116/141). Patients with insufficient biopsy were excluded from the study. There were no pathological findings in 100 of the biopsies and they constituted the normal histology group. 16 biopsies were considered as abnormal placental histology. Defective physiological change was seen in all abnormal biopsies. Apart from this, in three biopsies, there were acute atherosis, in another one there was increased extravillous trophoblasts, and in another one there were both acute atherosis and increased extravillous trophoblasts (Figures 1, 2, and 3).

Demographic and obstetric characteristics of cases enrolled in the study are shown in Table 1. Birth weight values were statistically significantly different (*P* < 0,05). There were no significant differences between two groups in other parameters.

Clinical disease was developed in six pregnancies. Four of them had preeclampsia; two of them had IUGR. In the normal histology group one fetus was diagnosed as IUGR. But the baby had additional anomalies (posterior urethral valve, prune belly syndrome) and died after birth. There were no preeclampsia cases in the normal histology group. Other IUGR case and preeclampsia cases were in the abnormal placental histology group. Notch was detected in these cases in either trimester. Each of these cases PI and RI values was >95th percentile.

The mean values and statistical evaluation of first and second trimester uterine artery pulsatility index (PI) and resistance index (RI) measurements are given in Table 2. Uterine artery RI and PI values were significantly different between two groups in second trimester. Bilateral notch ratio was significantly different between two groups in each trimester. Bilateral notch was found in all abnormal cases. Uterine artery PI and RI mean values were 1,42 and 0,67 in abnormal placental histology group. For normal histology group, they were 12, 1,02, and 0,56% in order (*P* < 0,01).

## 4. Discussion

Different methods for placental bed sampling were described in previous studies [21, 22]. Punch biopsy forceps, sharpened over forceps, and scissor were used for sampling in different studies. In these studies, criterion for sufficient biopsy was detection of trophoblasts or spiral arteries in the specimen. Gerretsen et al. showed that a single large biopsy had a higher rate of success compared to multiple biopsies [10]. It is not necessary to take biopsy deeper than few millimeters in the myometrium. In one study, it was shown that the number of interstitial trophoblasts was decreased dramatically deeper than 3 millimeters in the myometrium [23]. In previous studies, rates of successful biopsies were between 44 and 70% [22–24]. In our study, placental biopsies were taken during cesarean section after delivery and manual removal of placenta; one biopsy was taken in the central portion of the placental bed with 8 × 5 × 3 mm Kevorkian punch biopsy forceps. We achieved 82.2% success rate in this study. High success rate in this study may be due to use of standardized method by the one operator. We followed up 510 pregnancies in antenatal period, but 270 women were admitted to our clinic for delivery. In Istanbul, patients usually prefer private hospitals for vaginal delivery and they admit to university hospitals if cesarean delivery is mandatory. High cesarean ratio in our hospital could be explained by this condition.

Defective physiological change of spiral arteries is manifested by increased resistance and pulsatility index of uterine artery and branches. In preeclampsia and IUGR cases after the clinical disease is emerged there is correlation between Doppler studies and pathological findings in the placental bed biopsies [20, 25]. In IUGR cases with high pulsatility index and/or notching in Doppler assessment, there is significantly higher ratio of pathological findings in placental bed biopsies compared to the group that shows normal Doppler findings [20]. Also birth weight is associated with placental bed vascular pathologies [11]. But in our study there was no significant difference among two groups for birth weights.

Voigt and Becker showed that pathological uterine artery Doppler indices were correlated with defective placentation with 90% sensitivity and 95% specificity [13]. In a study, Lin et al. accepted S/D > 2,5 as pathological Doppler finding and they concluded that with 92,3% PPV abnormal uterine artery Doppler indices were correlated with defective trophoblast invasion in myometrium [26]. But in these studies Doppler assessments were made after preeclampsia had been diagnosed at term pregnancy. In a meta-analysis, Cnossen et al. concluded that use of PI and notching together is the best predictor for preeclampsia and IUGR [18]. In another meta-analysis, Kleinrouweler et al. found that the combination of Doppler ultrasound parameters PI or RI and bilateral notching significantly improves the prediction of preeclampsia based on the patient characteristics BMI and systolic blood pressure [27]. In the present study, bilateral notching was found in 45/100 of the normal histology group in first trimester and 12/100 of the normal histology group in second trimester. The ratio was 16/16 of the abnormal placental histology group (*P* < 0,001). In the normal histology group, mean value of PI was 1,02 ± 0,31 and mean value of RI was 0,56 ± 0,08 at second trimester. In the abnormal placental histology group, means were 1,42 ± 0,21 and 0,67 ± 0,05, respectively. There was statistically significant difference between two groups in terms of RI and PI measurements (*P* < 0,001). We concluded that there was statistically significant correlation between second trimester Doppler assessment and pathological findings in the placental bed biopsy. Among these parameters the most reliable one was detection of bilateral notch.

In two studies, uterine artery RI was evaluated in women attending clinics for termination of pregnancy in the first trimester. Their results indicated that decidual natural killer cells isolated from pregnancies with a high RI, which are at a higher risk of preeclampsia, were less able to chemoattract trophoblasts and less able to induce extravillous trophoblast outgrowth from the villi, failed to induce vascular apoptosis, and secreted fewer proapoptotic factors. Using uterine artery Doppler RI screening before cell isolation has, therefore, identified that decidual natural killer cells from high RI pregnancies have altered interactions with trophoblasts and impaired vessel remodelling, which may lead to poor placentation [28, 29]. Whitley et al. showed that first trimester extravillous trophoblasts from pregnancies with high uterine artery resistance are more sensitive to apoptotic stimuli [30]. It was shown that high resistance uterine artery Doppler indices are related to defective endovascular trophoblast invasion in the first trimester [31]. In our study, in first trimester, mean value of PI was 1,64 ± 0,45 and mean value of RI was 0,70 ± 0,08 in the normal group. Means were 1,91 ± 0,52 and 0,73 ± 0,1 in the abnormal placental histology group. There was no statistically significant difference between two groups.

In this study, sensitivity and specificity were higher than in previous studies which correlated Doppler results with clinical outcome. Pathological Doppler results indicate high resistance in uterine artery due to defective placentation. But clinical disease is multifactorial so, in some cases with pathological Doppler findings, preeclampsia and/or IUGR are not developed. Also samples belong to very small portion of whole placental bed. Therefore, biopsies might not reflect overall vascular histology of the placental bed [32]. Eleven clinically normal pregnancies in the abnormal placental histology group may be explained with diffusiveness of the pathology in the placental bed.

The fact that all biopsies were examined by the same pathologist with a well-defined standardized protocol greatly increases the internal validity of the results. Potential limitations must be acknowledged. First, the placenta is a large organ, and the accurate assessment of placental bed pathology is difficult with single biopsy. Second, the study groups were small in number. We could evaluate 116 cases from a cohort of 270 women. 129 women delivered vaginally and another 25 cases' bed biopsies were accepted as “insufficient biopsy.”

## 5. Conclusion

There are differences in sensitivity among studies which correlated Doppler velocimetry and preeclampsia. Because there are other factors that are effective in the emergence of clinical outcome. In this study, we found that there was statistically significant correlation between second trimester Doppler indices and placental bed biopsy findings independent of clinical course.

## Figures and Tables

**Table 1:** Demographic and clinical characteristics of the study population.

	Normal placental histology group (*n* = 100)	Abnormal placental histology group (*n* = 16)	*P* value
Age	27,4 ± 4,8	28,8 ± 5	0,461
Parity	1 (0–6)	0 (0–3)	0,499
Birth weight	**3243 (±417)**	**2788 (±822)**	**0,015**
Birth week	38 (35–41)	38 (32–40)	0,419

Values are expressed as median (range) or mean (standard deviation).

**Table 2:** Statistical evaluation of Doppler findings.

	Normal placental histology group (*n* = 100)	Abnormal placental histology group (*n* = 16)	*P* value
1.trimester PI	1,64 ± 0,45	1,91 ± 0,52	0,158
1.trimester RI	0,70 ± 0,08	0,73 ± 0,1	0,462
1.trimester notch	**%45**	**%100**	**<0,001**
2.trimester PI	**1,02 ± 0,31**	**1,42 ± 0,21**	**<0,001**
2.trimester RI	**0,56 ± 0,08**	**0,67 ± 0,05**	**<0,001**
2.trimester notch	**%12**	**%100**	**<0,001**

Values are expressed as mean ± standard deviation.

## Abbreviations

IUGR: Intrauterine growth retardation
AUC: Area under curve
PI: Pulsatility index
RI: Resistance index
BMI: Body mass index.
